# The activation of gene expression and alternative splicing in the formation and evolution of allopolyploid *Brassica napus*

**DOI:** 10.1093/hr/uhab075

**Published:** 2022-01-19

**Authors:** Mengdi Li, Meimei Hu, Yafang Xiao, Xiaoming Wu, Jianbo Wang

**Affiliations:** 1 State Key Laboratory of Hybrid Rice, College of Life Sciences, Wuhan University, Wuhan 430072, China; 2 Key Laboratory of Resource Biology and Biotechnology in Western China, Ministry of Education, College of Life Sciences, Northwest University, Xi’an, Shaanxi 710069, China; 3 Key Laboratory of Biology and Genetic Improvement of Oil Crops, Ministry of Agriculture, Oil Crops Research Institute of CAAS, Wuhan 430062, China

## Abstract

Allopolyploids contain two or more sets of subgenomes. To establish a compatible relationship between subgenomes, a series of gene expression changes has occurred in allopolyploids. What evolutionary changes have taken place in transcripts of *Brassica napus* during its early establishment and subsequent evolution is a fascinating scientific question. Here, we study this issue using a set of materials (natural and resynthesized *B. napus* and their progenitors/parents) and long-read RNA sequencing technology. The results showed that more genes were upregulated in resynthesized *B. napus* compared with its two parents, and more upregulated expressed genes were observed in natural *B. napus* than in resynthesized *B. napus*. The presence of upregulated genes in an organism may help it to adapt to the influence of “genomic shock” and cope with the natural environment. Isoforms are produced from precursor mRNAs by alternative splicing (AS) events, and more than 60% of the isoforms identified in all materials were novel, potentially improving the reference genome information for *B. napus*. We found that the isoform numbers and the numbers of genes potentially involved in AS and alternative polyadenylation increased in *B. napus* after evolution, and they may have been involved in the adaptation of plants to the natural environment. In addition, all identified isoforms were functionally annotated by searching seven databases. In general, this study can improve our overall understanding of the full-length transcriptome of *B. napus* and help us to recognize the significant changes in gene expression and isoform abundance that have occurred in allopolyploid *B. napus* during evolution.

## Introduction

Interspecific hybridization and polyploidization are considered to be the intrinsic powers behind the evolution of plant genomes [1]. Polyploidization or whole genome duplication (WGD) is a common phenomenon in the evolution of angiosperms [2]. A previous study showed that more than 70% of flowering plants are polyploids [3]. Polyploids, especially important crops such as wheat, cotton, and oilseed rape, have been extensively studied from genetics and evolution [4, 5] to genomics and gene function [6, 7]. Based on the composition of their chromosomes, polyploids can be divided into autopolyploids and allopolyploids. Allopolyploids are very common and highly adaptable to a variety of biotic and abiotic stresses [8], perhaps because genetic redundancy and heterozygosity are present in allopolyploids, resulting in new traits and improved adaptive capacity [9, 10]. Allopolyploids have undergone a “genomic shock” event [11] that leads to enlargement of the nucleus, rearrangement of the chromosomes, and genomic changes, ultimately resulting in the reprogramming of transcriptomes, proteomes, and metabolomes in organisms [12]. Gene expression changes are the molecular basis for the successful establishment and evolution of allopolyploids, and studies have found that gene expression changes are related to the adaptation of polyploids [13]. There are some obvious characteristics of gene expression changes in allopolyploids, such as non-additive gene expression and the activation and silencing of gene expression [14, 15].

Alternative splicing (AS) refers to the process of producing different mRNAs from one mRNA precursor (pre-mRNA) by different splicing methods, and it is ubiquitous in eukaryotes [16]. AS is an important mechanism for regulating gene expression and also a critical driving force for promoting the diversity of the transcriptome and proteome in organisms [17]. As a regulatory mechanism with a profound influence on organismal function, AS controls many different developmental processes and responses to various environmental conditions [18]. Although short-read sequencing technology (such as Illumina sequencing) can accurately detect the splice sites of AS events, it is challenging to infer the actual combinations of splice sites because computational assembly is required for short reads [19]. Full-length isoforms can be sequenced directly without assembly using long-read sequencing technology, and this methodology can directly provide reliable evidence for AS events [20]. Currently, long-read sequencing technologies include Pacific Biosciences single-molecule long-read sequencing (PacBio) and Oxford Nanopore Technologies sequencing (ONT) [20]. In contrast to traditional sequencing technologies, ONT directly sequences a single-stranded nucleic acid molecule through a nanopore by detecting real-time, single-molecule electrical signals [21]. The advantages of ONT include extremely long sequencing reads, low sequencing costs, and the portability of sequencing instruments. ONT provides opportunities for detailed analyses of genetic changes in allopolyploids. To date, long-read sequencing technology has been used to study AS in corn [19], cotton [18], and wheat [22], but there have been no in-depth studies on oilseed rape. Furthermore, alternative polyadenylation (APA) events of pre-mRNAs can generate multiple transcript isoforms to enhance the complexity and diversity of the transcriptome, thus regulating gene expression through multiple mechanisms in eukaryotes [23–26].

*Brassica napus* is an important oil crop with a wide area of cultivation. It is an allotetraploid species that contains two largely collinear genomes (A and C) donated by its diploid progenitors (*Brassica rapa* and *Brassica oleracea*) ~7500 years ago [27]. Resynthesized *B. napus* allopolyploids with known parents are excellent materials for detecting genomic changes during the early stages of *B. napus*formation [28]. Our study on resynthesized and natural *B. napus* can also provide clues about the genomic changes in allopolyploid *B. napus* over the long course of natural evolution. In this study, we performed long-read transcriptomic analysis of resynthesized *B. napus*, its two diploid parents, and natural *B. napus* to explore the possible molecular basis for the successful establishment and adaptation of allopolyploid *B. napus.*

## Results

### Transcriptome sequencing using Oxford Nanopore Technologies (ONT)

Leaf samples from natural allotetraploid *B. napus* (NAC), resynthesized *B. napus* (RAC), and its parents were harvested to build RNA libraries for third-generation sequencing, each with three biological replicates. The RNA samples used to build the libraries were of high quality (Table S1). A total of 113.54 million clean reads with high quality (mean quality values >9.2) from 12 RNA libraries were generated using ONT (Table 1). Then, the *in silico* “hybrid” (A_C) was constructed by mixing full-length reads from *B. rapa* and *B. oleracea* at a ratio of 1:1 as described in previous studies [1, 29]. The ratio of full-length read number to clean read number averaged about 77.7% in all samples (Table 1). According to the first alignment results, an average of 78.5%, 70.3%, 61.8%, and 71.0% of the clean reads from the samples of natural *B. napus*, resynthesized *B. napus*, *B. oleracea* (maternal parent, C), and *B. rapa* (paternal parent, A) were mapped to the *B. napus* genome [27], respectively (Table 1). The average GC content of the consensus reads in all samples was 43.3% (Table 1).

**Table 1:** Statistics of RNA-seq data for all samples

**Sample** ^**a**^	**Number of clean reads**	**Number of full-length reads**	**Number of consensus reads**	**Mean quality**	**FL/clean reads** ^**b**^ **(%)**	**Mapping genome ratio (%)**	**GC content (%)**
A1	8 053 769	6 399 073	59 413	9.6	79.5	75.80	43.31
A2	9 792 200	7 421 972	64 491	9.5	75.8	65.47	42.60
A3	7 924 047	6 420 927	53 684	9.6	81.0	71.77	43.25
C1	15 766 578	10 163 265	83 749	9.3	64.5	55.15	42.10
C2	14 440 698	8 618 486	83 397	9.2	59.7	58.97	42.27
C3	7 607 359	5 972 210	58 999	9.7	78.5	71.28	43.87
RAC1	8 664 816	6 699 685	65 629	9.4	81.8	68.84	43.75
RAC2	9 305 060	6 531 206	65 041	9.3	83.2	69.63	43.13
RAC3	7 226 119	7 262 352	69 989	9.4	83.3	72.54	44.10
NAC1	8 191 696	7 041 196	67 755	9.4	81.3	71.07	43.80
NAC2	7 852 928	7 397 637	69 475	9.5	79.5	82.66	43.87
NAC3	8 716 848	6 072 581	64 014	9.4	84.0	81.77	43.83

A: *B. rapa*; C: *B. oleracea*; RAC: resynthesized *B. napus*; NAC: natural *B. napus*

a 1, 2, and 3 represent three biological replicates

b FL/clean reads represents the ratio of full-length read number to clean read number

### Analysis of expression levels and differentially expressed genes (DEGs)

**Figure 1:**
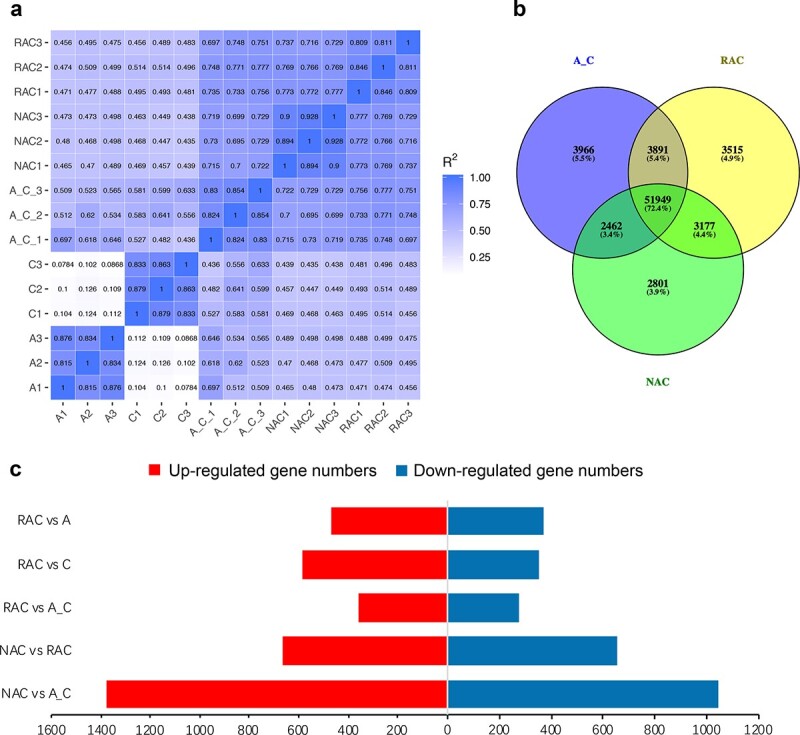
Expression and differential expression of identified genes. **a** Pearson correlation coefficients between three biological replicates. **b** Venn diagram of the number of expressed genes in the *in silico* “hybrid”, resynthesized *B. napus*, and natural *B. napus*. **c** Differentially expressed genes (DEGs) in different comparison groups; the numbers of up- and downregulated genes in each comparison group are represented by red and green bars in the histogram. A: *B. rapa*; C: *B. oleracea*; A_C: *in silico* “hybrid”; RAC: resynthesized *B. napus*; NAC: natural *B. napus*.

The gene expression correlations between the three biological replicates of all selected species were high, and the Pearson correlation coefficient (*R*) values were all over 0.8 (Figure 1a). The transcripts per million reads (TPM) method was used to normalize the gene expression levels, and genes with TPM > 0 were considered to be expressed in the samples in this study. We randomly selected nine genes and verified their expression levels by real-time quantitative PCR (qRT-PCR). The results showed that ONT sequencing technology was reliable for gene expression detection (Figure S1). A total of 29 352, 33 507, 62 268, 62 532, and 60 389 genes were expressed in *B. rapa*, *B. oleracea*, the *in silico* “hybrid”, resynthesized *B. napus*, and natural *B. napus*, respectively. As expected, most of the genes (51 949, 72.4%, Figure 1b) were co-expressed in the two allotetraploid *B. napus* and the *in silico* “hybrid”. The expression of 6428 genes (3966 + 2462, Figure 1b) was silenced, and 6692 genes (3515 + 3177, Figure 1b) were activated in resynthesized *B. napus* compared with the *in silico* “hybrid”. Similarly, the expression of 7406 genes (3515 + 3891, Figure 1b) was silenced, and 5263 genes (2462 + 2801, Figure 1b) were activated in natural *B. napus* compared with resynthesized *B. napus*. A total of 839 and 937 differentially expressed genes (DEGs) were detected in the comparison groups of resynthesized *B. napus* with its two parents, respectively, and more of these DEGs were upregulated in resynthesized *B. napus* (Figure 1c), indicating that more gene expression may be activated during the formation of resynthesized *B. napus*. A total of 637 genes displayed significant differential expression between resynthesized *B. napus* and the *in silico* “hybrid”, 360 of which (173 in the A_n_ subgenome and 181 in the C_n_ subgenome) were upregulated and 277 of which (156 in the A_n_ subgenome and 120 in the C_n_ subgenome) were downregulated in resynthesized *B. napus* (Figure 1c). In addition, 1317 genes were differentially expressed between natural and resynthesized *B. napus*, 664 of which (385 in the A_n_ subgenome and 276 in the C_n_ subgenome) were upregulated and 653 of which (287 in the A_n_ subgenome and 364 in the C_n_ subgenome) were downregulated in natural *B. napus* (Figure 1c). The above results implied that the expression of genes on the C_n_ subgenome may be more likely to be activated in resynthesized *B. napus* during the hybridization and polyploidization process, whereas the expression of genes on the A_n_ subgenome may be more likely to be activated in natural *B. napus* during the natural evolutionary process.

### Analysis of non-additively expressed genes

**Figure 2:**
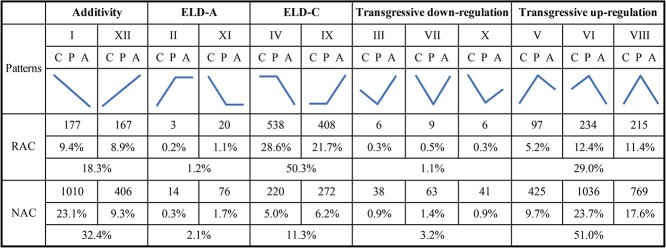
The 12 patterns of additively and non-additively expressed genes in resynthesized *B. napus* (RAC) and natural *B. napus* (NAC). C, P, and A indicate *B. oleracea*, progenies, and *B. rapa*, respectively.

Non-additively expressed genes, including genes that show expression-level dominance (ELD) and transgressive regulation, have been analyzed in a growing number of studies on allopolyploids. We performed an analysis of non-additively expressed genes, and the expressed genes in the two *B. napus* allopolyploids were classified into 12 categories according to a previous study [30]. The 12 categories could be further grouped into 3 categories: additive, ELD, and transgressive expression. Additive expression occurs when the expression level of a gene in the progenies is close to the median value of its expression in the two parents/progenitors. ELD occurs when the gene expression level in the progenies only approaches its expression level in one parent/progenitor. Transgressive expression occurs when the expression level of a gene in the progenies is higher/lower than that in both parents/progenitors. Overall, 18.3%, 51.5%, and 30.1% of genes exhibited additive, ELD, and transgressive expression patterns in resynthesized *B. napus* (Figure 2). Moreover, a total of 32.4%, 13.4%, and 54.2% of genes showed additive, ELD, and transgressive expression patterns in natural *B. napus* (Figure 2). There were fewer additively expressed genes than non-additively expressed genes in this study, consistent with previous studies [28, 31]. More genes displayed the ELD-C pattern rather than the ELD-A pattern, and more transgressively upregulated genes than downregulated genes were observed in both types of *B. napus* (Figure 2). The number of transgressively upregulated genes clearly increased during the subsequent evolution of *B. napus*, indicating that transgressively upregulated genes may help plants to cope with a variety of natural environments.

### Analysis of AS and splicing isoforms of pre-mRNAs

At present, the complexity of AS events in allotetraploid *B. napus* remains largely unsolved because the two subgenomes are highly similar, increasing the difficulty of exploration and analysis using second-generation sequencing assembly methods. An important feature of third-generation transcriptome sequencing is that full-length transcripts can be obtained without assembly, and the AS events of genes can therefore be accurately detected. Based on obtaining high-quality, full-length transcripts, AS events were systematically analyzed in the samples. Different isoforms are produced from pre-mRNAs by AS events, and we identified isoforms first.

**Figure 3:**
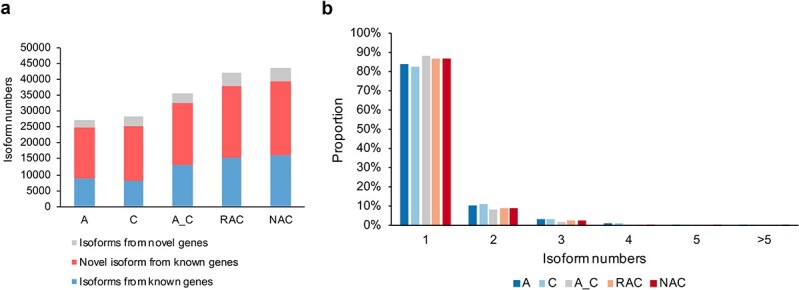
Information on the isoforms identified in *B. rapa* (A), *B. oleracea* (C), the *in silico* “hybrid” (A_C), resynthesized *B. napus* (RAC), and natural *B. napus* (NAC). **a** Numbers of three types of isoforms (isoforms from known genes, novel isoforms from known genes, and isoforms from novel genes). **b** The proportions of genes with different numbers of isoforms.

Three types of isoforms (isoforms from known genes, novel isoforms from known genes, and isoforms from novel genes) were identified by annotating consensus reads (Table S2). As shown in Figure 3a, the number of novel isoforms (including novel isoforms from known genes and isoforms from novel genes) in each species accounted for more than 60% of the total number of isoforms. This result indicates that many novel isoforms were identified by third-generation transcriptome sequencing, increasing our understanding of the complexity of the *B. napus* reference genome and potentially improving the information of the reference genome. Information on all identified isoforms is provided in Dataset S1. In addition, isoforms from novel genes accounted for a small proportion (Figure 3a), indicating that the reference genome is relatively complete. The largest proportion of isoforms were novel isoforms from known genes (Figure 3a), indicating that multiple isoforms of the same gene in the existing genome data are incomplete and require further supplementation. The total number of isoforms in the resynthesized allotetraploid *B. napus* was higher than that in the *in silico* “hybrid”, and the total number of isoforms was slightly higher in natural tetraploid *B. napus* than in resynthesized *B. napus* (Figure 3a). In all samples, more than 80% of the genes had only a single isoform, and about 10% of the genes produced two isoforms (Figure 3b).

**Figure 4:**
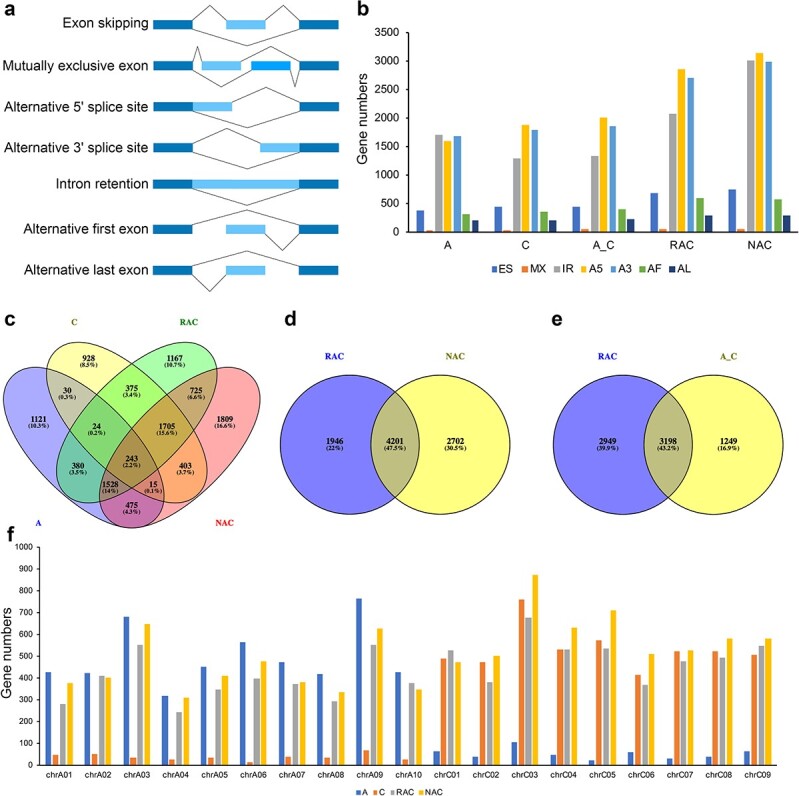
Characterization of alternative splicing (AS) events. **a** Visualization of seven AS modes. **b** Numbers of different types of AS in *B. rapa* (A), *B. oleracea* (C), the *in silico* “hybrid” (A_C), resynthesized *B. napus* (RAC) and natural *B. napus* (NAC). **c** Venn diagram of known genes with AS events among the four species (A, C, RAC, and NAC). **d** Venn diagram of known genes with AS events in RAC and NAC. **e** Venn diagram of known genes with AS events in RAC and A_C. **f** The numbers of genes with AS events on different chromosomes in A, C, A_C, RAC, and NAC. ES: exon skipping; MX: mutually exclusive exon; IR: intron retention; A5: alternative 5′ splice site; A3: alternative 3′ splice site; AF: alternative first exon; AL: alternative last exon.

To verify whether the AS events detected by ONT sequencing technology were accurate, we selected five genes for PCR amplification experiments, and the results showed that the AS events detected by ONT sequencing technology were credible (Figure S2). A total of 5903 AS events from 4013 gene loci were detected in *B. rapa*, 6008 AS events from 4020 gene loci in *B. oleracea*, 6349 AS events from 4682 gene loci in the *in silico* “hybrid”, 9296 AS events from 6480 gene loci in resynthesized *B. napus*, and 10 820 AS events from 7327 gene loci in natural *B. napus*. The total number of AS events was higher in *B. oleracea* than in *B. rapa*, consistent with the results of isoform identification (the number of isoforms was greater in *B. oleracea* than in *B. rapa*, Figure 3a). There were significantly more AS events in resynthesized *B. napus* than in the *in silico* “hybrid” (Student’s *t*-test, *P* value <0.001), which may have helped resynthesized *B. napus* to cope with the “genomic shock” caused by the fusion of two genetic materials in the nucleus during hybridization. Significantly more AS events occurred in natural *B. napus* than in resynthesized *B. napus* (Student’s *t*-test, *P* value <0.001), which may have helped natural *B. napus* to adapt to the natural environment during the long evolutionary process. All AS events were divided into seven types (Figure 4a): exon skipping (ES), mutually exclusive exon (MX), alternative 5′ splice site (A5), alternative 3′ splice site (A3), intron retention (IR), alternative first exon (AF), and alternative last exon (AL). In natural *B. napus*, resynthesized *B. napus*, the *in silico* “hybrid”, and *B. oleracea*, genes with A5 events accounted for the largest proportion, followed by genes with A3 and IR events (Figure 4b). By contrast, in *B. rapa*, genes with IR events accounted for the largest proportion, followed by genes with A3 and A5 events (Figure 4b). In all samples, genes with MX events were the least common, and genes with three types of event (A5, A3, and IR) accounted for an overwhelming proportion (over 80%) of all AS events (Figure 4b), indicating that these three types of AS event were most common in natural *B. napus*, resynthesized *B. napus*, and its parents. There were significantly more genes with IR events in natural *B. napus* than in resynthesized *B. napus* (Student’s *t*-test, *P* value <0.001, Figure 4b), indicating that the increased IR events may have helped natural *B. napus* to adapt to the natural environment during the evolutionary process.

A Venn diagram was drawn using a list of pre-mRNAs with AS events in the four species (Figure 4c–e). We found that 243 pre-mRNAs had AS events in all four species, and 1809 pre-mRNAs had AS events only in natural *B. napus* (the largest number in the four species, Figure 4c). More than half (52.5%) of the pre-mRNAs did not maintain the AS events during the subsequent evolution of *B. napus* (Figure 4d), suggesting that the AS events of the pre-mRNAs changed greatly, which may have contributed to the adaptation of natural *B. napus* to the environment. There were 2949 (about 39.9%) pre-mRNAs with AS events only in resynthesized *B. napus* and 1249 (about 16.9%) pre-mRNAs with AS events only in the *in silico* “hybrid” (Figure 4e), suggesting that more pre-mRNAs with AS events in resynthesized *B. napus* may be helpful for coping with “genomic shock” during the early establishment of *B. napus*. By counting the number of pre-mRNAs with AS events from genes on each chromosome of the four species, we found that there were many pre-mRNAs with AS events from genes on chromosome 3 of both two subgenomes (A_n_ and C_n_, Figure 4f), indicating that active AS events occurred on this homologous chromosome in the four species. The highest number of pre-mRNAs with AS events was detected from genes on chromosome C03 in the two allotetraploid *B. napus* (Figure 4f). The number of pre-mRNAs with AS events from genes on chromosomes A03 and A09 decreased after the formation of allotetraploid *B. napus* (Figure 4f). During the early establishment and subsequent evolution of *B. napus*, the number of pre-mRNAs with AS events from genes on chromosome C05 first decreased and then increased (Figure 4f). The pre-mRNAs with only one AS event in each sample accounted for at least 85% of all pre-mRNAs (Table S3).

### Functional annotation of isoforms

All identified isoforms were functionally annotated by searching seven databases, and more than 99% of the isoforms in each species were annotated by at least one database (Table 2). The annotation results from the NR database showed that the largest number of isoforms was distributed in *B. napus* (over 70% in all selected species) and that more than 94% of the isoforms were annotated in three species (*B. napus*, *B. rapa*, and *B. oleracea*) (Figure S3a). The GO annotation showed that all identified isoforms could be divided into three groups, biological processes (BP), cellular components (CC), and molecular functions (MF) (Figure S3b–e). Isoforms from all four species were enriched in the same GO terms. Specifically, the GO terms enriched in the BP category included mainly cellular process, metabolic process, and single-organism process, the GO terms enriched in the CC category included mainly cell and cell part; and the GO terms enriched in the MF category included mainly binding and catalytic activity in all four selected species (Figure S3b–e). According to the results of the COG annotation, all isoforms were annotated to 25 function classes, and the top three classes with the largest numbers of genes included “General function prediction”, “Posttranslational modification, protein turnover, chaperones”, and “Signal transduction mechanisms” (Figure S3f). All annotation results for isoforms in the four species are provided in Dataset S2.

**Table 2:** Numbers of functionally annotated isoforms

**Annotation database**	**Isoform number**			
	**A**	**C**	**RAC**	**NAC**
NR	26 295	27 068	40 813	42 650
Swiss-Prot	21 519	21 647	33 356	34 864
KEGG	25 881	26 287	39 965	41 766
COG	16 242	16 631	25 057	26 277
GO	17 861	17 405	28 456	29 606
NT	27 081	28 314	41 875	43 608
Pfam	17 861	17 405	28 456	29 606
All annotated	27 101	28 360	41 927	43 639
All analyzed	27 136	28 449	42 005	43 692
Annotation rate	99.9%	99.7%	99.8%	99.9%

### Analysis of alternative polyadenylation (APA)

**Figure 5:**
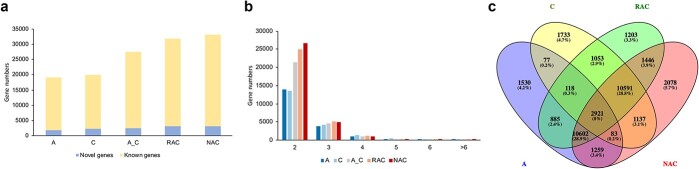
Information on alternative polyadenylation (APA) events. **a** Numbers of known genes (yellow) and novel genes (blue) that encoded pre-mRNAs with two or more poly(A) sites. **b** Numbers of genes that encoded pre-mRNAs with different numbers of poly(A) sites. **c** Venn diagram of genes with potential APA events in A, C, RAC, and NAC. A, *B. rapa*; C, *B. oleracea*; A_C, *in silico* “hybrid”; RAC, resynthesized *B. napus*; NAC, natural *B. napus*.

Pre-mRNAs transcribed from 19 216, 19 940, 31 804, and 33 152 genes contained two or more poly(A) sites in *B. rapa*, *B. oleracea*, resynthesized *B. napus*, and natural *B. napus*, respectively (Table 3, Dataset S3). These genes are potentially responsible for APA events. More than 88.8% of these genes are known genes in selected species (Table 3, Figure 5a). Most genes (75% on average) that encoded pre-mRNAs contained two poly(A) sites in all four species (Figure 5b). The number of genes that encoded pre-mRNAs with two poly(A) sites in the two allotetraploids was about 1.6 times that of the two diploids, whereas the number of genes that encoded pre-mRNAs with three or more poly(A) sites did not differ significantly among the species (Figure 5b). The number of genes that encoded pre-mRNAs with two or more poly(A) sites was significantly higher in resynthesized *B. napus* than in the *in silico* “hybrid” (Student’s *t*-test, *P* value <0.001, Figure 5a, 5b), indicating that more APA events in pre-mRNAs of resynthesized *B. napus* may help it to cope with a series of effects brought about by hybridization and polyploidization. Similarly, the number of genes that encoded pre-mRNAs with two or more poly(A) sites was significantly higher in natural *B. napus* than in resynthesized *B. napus* (Student’s *t*-test, *P* value <0.001, Figure 5a, 5b), indicating that the increase in APA events in pre-mRNAs may help the plant to cope with changes in the natural environment. To explore the evolutionary changes in poly(A) sites of pre-mRNAs, we performed an overlap analysis for pre-mRNAs with two or more poly(A) sites in all selected species. Most poly(A)-containing pre-mRNAs were common between *B. rapa*/*B. oleracea* and resynthesized *B. napus* (83.1% in *B. rapa*, 82.9% in *B. oleracea*, Figure 5c), and about 88.7% of the poly(A)-containing pre-mRNAs were common between resynthesized and natural *B. napus*. In addition, although 3259 poly(A)-containing pre-mRNAs were specific to resynthesized *B. napus*, the other 4557 poly(A)-containing pre-mRNAs were specific to natural *B. napus* (Figure 5c). These results suggested a possible important role for APA events in the evolution of *B. napus*.

**Table 3:** Numbers of genes that encoded pre-mRNAs with two or more poly(A) sites

**Sample**	**Known genes**	**Novel genes**	**Total**
A	17 475	1741	19 216
C	17 713	2227	19 940
A_C	25 171	2387	27 558
RAC	28 819	2985	31 804
NAC	30 117	3035	33 152

### Analysis of transcription factor genes

**Figure 6:**
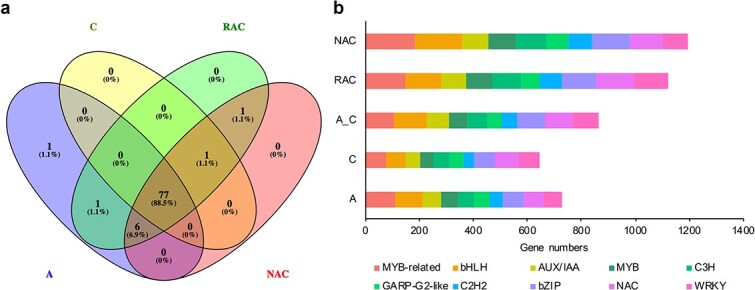
Information on identified transcription factors (TFs). **a** Venn diagram of identified TF families in *B. rapa* (A), *B. oleracea* (C), resynthesized *B. napus* (RAC), and natural *B. napus* (NAC). **b** The top 10 TF families in A, C, the *in silico* “hybrid” (A_C), RAC, and NAC.

Transcription factor (TF) genes play critical regulatory roles in plant growth and development. A total of 1596 putative TF gene members from 85 TF families, 1367 members from 78 TF families, 1837 members from 81 TF families, 2450 members from 86 TF families, and 2656 members from 85 TF families were identified in *B. rapa*, *B. oleracea*, the *in silico* “hybrid”, resynthesized *B. napus*, and natural *B. napus*, respectively (Dataset S4). Seventy-seven TF gene families were common to all selected species (Figure 6a). TF genes were more diverse and abundant in *B. rapa* than in *B. oleracea* (Figure 6a, Dataset S4). Compared with genes in resynthesized *B. napus*, more genes in natural *B. napus* were identified as TF genes (Dataset S4), indicating that some TF genes may be activated during the natural evolution of *B. napus*. Compared with the *in silico* “hybrid”, both resynthesized *B. napus* and natural *B. napus* had richer and more abundant TF genes (Dataset S4), indicating that allotetraploids produced by hybridization and polyploidization may tend to retain more TF genes, which may help the plants cope with the impact of hybridization, polyploidization, and various aspects of the natural environment. The top 10 TF gene families in all samples were MYB-related, bHLH, NAC, bZIP, AUX/IAA, WRKY, MYB, C_3_H, GARP-G2-like, and C_2_H_2_ (Figure 6b). The total number of top-10 TF family genes was higher in the allotetraploids than in the diploids (Figure 6b). After the formation of resynthesized *B. napus* by hybridization and polyploidization of *B. rapa* and *B. oleracea*, 79.2% (61 out of 77) TF families experienced partial loss or expression silencing of TF genes. Among these 61 TF families, 42 (68.9%) may have experienced expression activation or an increase in the number of TF genes in natural *B. napus* (Dataset S4). In addition, to further explore how many TF genes exhibit AS events, we performed a joint analysis of TF genes and pre-mRNAs with AS events. The results showed that 254, 213, 267, 389, and 444 pre-mRNAs encoded by TF genes exhibited AS events in *B. rapa*, *B. oleracea*, the *in silico* “hybrid”, resynthesized *B. napus*, and natural *B. napus*, respectively. It can be seen that the number of TF genes with AS events increased during the formation and evolution of allopolyploid *B. napus*, and this may be related to the enhancement of plant environmental adaptability.

## Discussion

Allotetraploid *B. napus*, an important oil crop, is a critical model plant for studying the scientific problems related to hybridization and polyploidization [28, 31]. In the last decade, high-throughput sequencing technology has deepened our understanding of the transcriptome landscape in many organisms [18]. Many interesting scientific questions, including the acquisition and loss of gene families, changes in gene expression, the regulation of gene expression, and expression level dominance of homologous gene pairs, have been studied in succession in allotetraploid *B. napus* using this sequencing technology [28, 31–35]. All these studies used second-generation sequencing technology, which has some disadvantages associated with the need to assemble the sequencing results. For example, full-length transcripts cannot be obtained accurately, and AS and APA events can therefore not be identified accurately. Moreover, homologous genes in polyploids often encode highly similar isoforms that can increase the complexity of the transcriptome [18, 36]. Thus, it is a great challenge to carry out systematic studies on AS and APA in allopolyploids. The recent emergence of third-generation long-read sequencing technology provides an excellent opportunity to resolve this conundrum. Four materials, natural *B. napus*, resynthesized *B. napus*, and their parents, were selected for this study. We hope to use this set of materials to study evolutionary changes in *B. napus* during its two important stages (early formation and natural evolution for about 7500 years [27]). Given that we do not know and cannot obtain the true parents of natural *B. napus*, we are unable to fully reproduce their evolution over the past 7500 years. Therefore, the materials selected for this study can only reflect these evolutionary changes to a certain extent. Despite these limitations, this set of materials may still be one of the most suitable options available for addressing the scientific concerns of this study. Here, we systematically analyzed a series of genetic changes in the transcriptome of *B. napus* during its early formation and long natural evolution using long-read RNA sequencing technology, aiming to improve our overall understanding of the full-length transcriptome of *B. napus*.

## Improvement of reference genome information for *B. napus*

The published genome sequence of allotetraploid *B. napus* has aroused widespread interest among genome biologists and breeders [27]. This genome sequence was assembled from the results of second-generation sequencing technology, and its annotations are not quite complete, preventing researchers from accurately analyzing gene structure and function in *B. napus* using only the genome data. In this study, more than 63% of the isoforms were identified as novel in both resynthesized and natural *B. napus* by mapping to the reference genome (Figure 3a). It can be seen that many novel isoforms can be identified by third-generation transcriptome sequencing technology, improving the reference genome information for *B. napus* and deepening our understanding of the complexity of the reference genome. In addition, the AS analysis, APA analysis, and functional annotation results from seven databases in this study provide good resources for the genome annotation of allotetraploid *B. napus*. Without accurate annotation of isoforms, the functional differences among isoforms cannot be determined [18]. The identification of genes with AS will contribute to functional genomics in *B. napus*. Different transcript isoforms can be produced from genes through AS events in eukaryotes. In the current study, a total of 9296 AS events from 6480 gene loci were detected in resynthesized *B. napus* and 10 820 AS events from 7327 gene loci in natural *B. napus* using ONT sequencing technology. Pre-mRNAs with A5 events represented the largest proportion, followed by pre-mRNAs with A3 and IR events, and these three types of AS events accounted for an overwhelming proportion (over 80%) of all AS events (Figure 4b) in both natural and resynthesized *B. napus*. Studies on AS events in rice, soybean, corn, and cotton have shown that IR events also account for a large proportion [18, 37–39]. AS events greatly increased the complexity of gene transcription in allotetraploid *B. napus*.

## Activation of gene expression in allotetraploid *B. napus* may contribute to its environmental adaptation

The increase in genes and genome dose in allopolyploids will cause a “genomic shock” event after hybridization and polyploidization [11]. To establish a compatible relationship between the divergent genomes and thus alleviate problems such as genomic instability and chromosome imbalance, some genetic and epigenetic changes will occur in plants, leading to a series of “omics” changes [12, 31]. Therefore, how gene expression changes to coordinate multiple sets of divergent genomes and regulate their interactions in *B. napus* during the early stage of its formation and subsequent natural evolution has been a fascinating scientific question. Hybridization activated the expression of many genes, and the DEGs between hybrids and their parents were upregulated rather than downregulated in broccoli hybrids [40]. In this study, more genes were activated than silenced during hybridization and polyploidization to form resynthesized *B. napus* (Figure 1b), and more DEGs between resynthesized *B. napus* and its two parents displayed higher expression in resynthesized *B. napus* (Figure 1c). Moreover, the results of non-additive expression analysis also showed that there were more transgressively upregulated genes than downregulated genes in resynthesized *B. napus* (Figure 2). The above results suggest that the activation or upregulation of more gene expression in resynthesized *B. napus* may help it adapt to the influence of “genomic shock” brought about by the fusion of multiple sets of chromosomes in the nucleus. In addition, more DEGs between natural and resynthesized *B. napus* were upregulated than downregulated in natural *B. napus* (Figure 1c), and more transgressively upregulated than downregulated genes were observed in natural *B. napus* (Figure 2), implying that more genes with upregulated expression may help *B. napus* to cope with a variety of natural environments.

## More splicing isoforms in allotetraploid *B. napus* may contribute to its environmental adaptation

Polyploidy is a ubiquitous phenomenon that dominates the genome evolution of all flowering plants [11, 12, 15, 41–44]. Polyploids have the advantage of additional genetic materials required for plant evolution, adaptation, and domestication [15, 41, 43–46]. In general, polyploidy leads to an increase in the size of organisms or cells, which may affect the transcriptome size or transcript abundance [44]. In plants, the number of AS events affects the abundance of transcripts. The AS events of pre-mRNAs are an important part of the plant immune response, and AS may also be involved in adaptation to the environment [47]. This previous study also noted that some genes show changes only in their splicing isoform ratios without changing their total mRNA levels in response to biological interactions [47]. Therefore, the abundance of splicing isoforms may have a great influence on plant environmental adaptation. In addition, APA events of pre-mRNAs also affect transcript abundance. In this study, we found that the number of splicing isoforms, the number of pre-mRNAs with AS events, the number of pre-mRNAs with APA events, and the number of TF genes all increased successively in the *in silico* “hybrid”, resynthesized *B. napus*, and natural *B. napus*. Thus, although some gene loss events occurred, these isoform numbers still increased during both evolutionary processes in *B. napus* (early establishment and subsequent evolution), which may imply a potential relationship between the increase in isoform numbers and the adaptive evolution of *B. napus*.

## Materials and methods

### Plant materials

Seeds of four plant materials, natural allotetraploid *B. napus* (cv. Darmor, 2n = 4x = 38, A_n_A_n_C_n_C_n_), resynthesized allotetraploid *B. napus* (cv. HC-2, 2n = 4x = 38, A_n_A_n_C_n_C_n_), and its parents, wild *B. oleracea* (cv. 3YS013, 2n = 18, C_o_C_o_, maternal) and *B. rapa* (cv. 9JC002, 2n = 18, A_r_A_r_, paternal), were kindly provided by the Oil Crops Research Institute, Chinese Academy of Agricultural Sciences, China. The resynthesized *B. napus* is an allotetraploid created using techniques including hybridization, embryo rescue, and chromosome doubling. Plump seeds were germinated on culture dishes covered with a double layer of filter paper moistened with double-distilled water (ddH_2_O) in a light incubator (model HP250G-C, 23°C, 16 h/8 h day/night). Nutritive soil and vermiculite were evenly mixed in a 1:1 ratio, the soil was moistened with half-strength Hoagland’s nutrient solution (pH 5.8), and the moist soil was placed into plastic pots for subsequent use. When the cotyledons of plant materials had fully unfolded, they were transferred to the prepared pots (two seedlings per pot) and placed in a light incubator. The plants were watered regularly with half-strength Hoagland’s nutrient solution (pH 5.8). Young leaves of 5-week-old plants were harvested and immediately frozen in liquid nitrogen for RNA extraction. Three biological replicates were used in this study.

### Library preparation and transcriptome sequencing

Total RNA from each sample was extracted using the RNAprep Pure Plant Kit (Tiangen, China) according to the manual. RNA samples were assessed by four methods. First, agarose gel electrophoresis (1% gel concentration, 180 V, 16 min) was used for the preliminary detection of RNA samples to determine the extent of RNA degradation. The OD_260/280_ ratio of RNA samples was then measured using a NanoDrop spectrophotometer (Thermo Fisher) to detect RNA purity. The RNA concentration was accurately quantified using a Qubit fluorometer (Invitrogen). Finally, RNA integrity was accurately measured with the Agilent 2100 Bioanalyzer system. RNA samples that passed all quality checks were used for subsequent experiments. The Oxford Nanopore Technologies kit (SQK-PCS109) was used for full-length cDNA library preparation. Sequencing was performed on the PromethION 24 platform using flow cells (PAE33370). Guppy software (Oxford Nanopore) was used for base calling of sequences. The NanoFilt tool in the Nanopack package was used to filter the sequences (length > 100, quality > 7) and obtain clean data.

### Construction of the *in silico* “hybrid”

Full-length reads from samples of *B. rapa* and *B. oleracea* were mixed in a 1:1 ratio to form an *in silico* “hybrid” [1, 29]. If the numbers of full-length reads in the two samples were different, reads were extracted based on the smaller number. There were three biological replicates for each species, and three *in silico* “hybrids” were therefore constructed.

### Full-length read identification and transcriptome alignment

Clean reads were analyzed using the Pinfish process. First, Pychopper (https://hpc.nih.gov/apps/pychopper.html) was used to identify full-length reads. Minimap2 was then used to map the full-length reads to the reference genome (*B. napus* genome v5, http://www.genoscope.cns.fr/brassicanapus/data/), and the alignment results were clustered. Minimap2 was used to align these clustering results to the reference genome again to obtain consensus reads, and the consensus reads were annotated using GffCompare software to identify three types of isoforms (isoforms of known genes, novel isoforms of known genes, and isoforms of novel genes). Gene expression levels were normalized using the TPM (transcripts per million reads) method.

### Analysis of AS and TF genes

SUPPA software [48] was used to analyze AS events. Venn diagrams were drawn using an online site (http://bioinfogp.cnb.csic.es/tools/venny/index.html). The PlantTFDB database [49] was used for TF gene analysis.

### Functional annotation of isoforms

Seven public databases were used for functional annotation of transcripts, including the NR (Non-Redundant Protein Database, https://ftp.ncbi.nlm.nih.gov/blast/db/), GO (Gene Ontology, http://geneontology.org/), COG (Cluster of Orthologous Groups of proteins, http://www.ncbi.nlm.nih.gov/COG/), KEGG (Kyoto Encyclopedia of Genes and Genomes, http://www.genome.jp/kegg/), Pfam (http://pfam.xfam.org/), SwissProt (http://www.ebi.ac.uk/uniprot/) and NT (Nucleotide Sequence Database, https://ftp.ncbi.nlm.nih.gov/blast/db/) databases.

### Analysis of APA and differentially expressed genes (DEGs)

The TAPIS process [26] was used with default parameters to predict variable adenosylation sites in transcripts. This process predicts poly(A) sites based on the comparison results of consensus sequences and reference genomes. DESeq2 [50] was used to identify DEGs, and genes with |log_2_(fold change)| ≥ 1 and padj (adjusted *P* value) ≤ 0.001 were considered to be DEGs in this study. Expression levels of all genes in the two allopolyploids were compared with the mid-parent value (MPV, half of the sum of the TPM values of both parents) using DESeq2, and genes with padj ≤ 0.05 were considered to be non-additively expressed. Furthermore, 12 gene expression patterns were summarized according to a previous study [30].

### qRT-PCR and PCR amplification experiments

First-strand complementary DNA (cDNA) was synthesized with reverse transcriptase (M-MLV RT, Promega, USA) using prepared, quality-checked RNA samples following the manufacturer’s instructions. The cDNA was then diluted 10-fold to serve as the template for qRT-PCR, which was performed using the ABI StepOnePlus Real-Time PCR System (Applied Biosystems, USA) according to the manufacturer’s instructions. *ACT2/7* was selected as the internal control gene to standardize the results. The relative gene expression levels were determined by the 2^−ΔΔCt^ method. The gene-specific primers were designed with Primer 5 software, and the primer sequences for qRT-PCR are listed in Table S4. The common PCR amplification experiment was performed using diluted cDNA and TaqMix (Tiangen, Beijing, China). The primer sequences used for PCR amplification are listed in Table S5.

## Acknowledgements

This work was supported by the National Natural Science Foundation of China (31970241).

## Author contributions

JW and ML conceived and designed the study. ML performed the bioinformatics analyses. ML wrote the manuscript. XW proposed some critical suggestions for the study and revised the manuscript. XW provided the experimental materials. ML, MH, and YX were responsible for planting materials. All authors read and approved the final manuscript.

## Data availability

The data that support the findings of this study are openly available at the NCBI Sequence Read Archive (SRA) database under accession numbers SRR13302173–SRR13302184.

## Conflict of interest

The authors declare that they have no conflict of interest.

## Supplementary data

Supplementary data are available at *Horticulture Research Journal* online.

## Supplementary Material

Web_Material_uhab075
