# Supplementary material for: The activation of gene expression and alternative splicing in the formation and evolution of allopolyploid Brassica napus
Source: Hortic Res. 2022 Jan 19;9:uhab075. doi: 10.1093/hr/uhab075 (PMC8923814; doi:10.1093/hr/uhab075)
Supplement: Web_Material_uhab075 [file web_material_uhab075.docx]

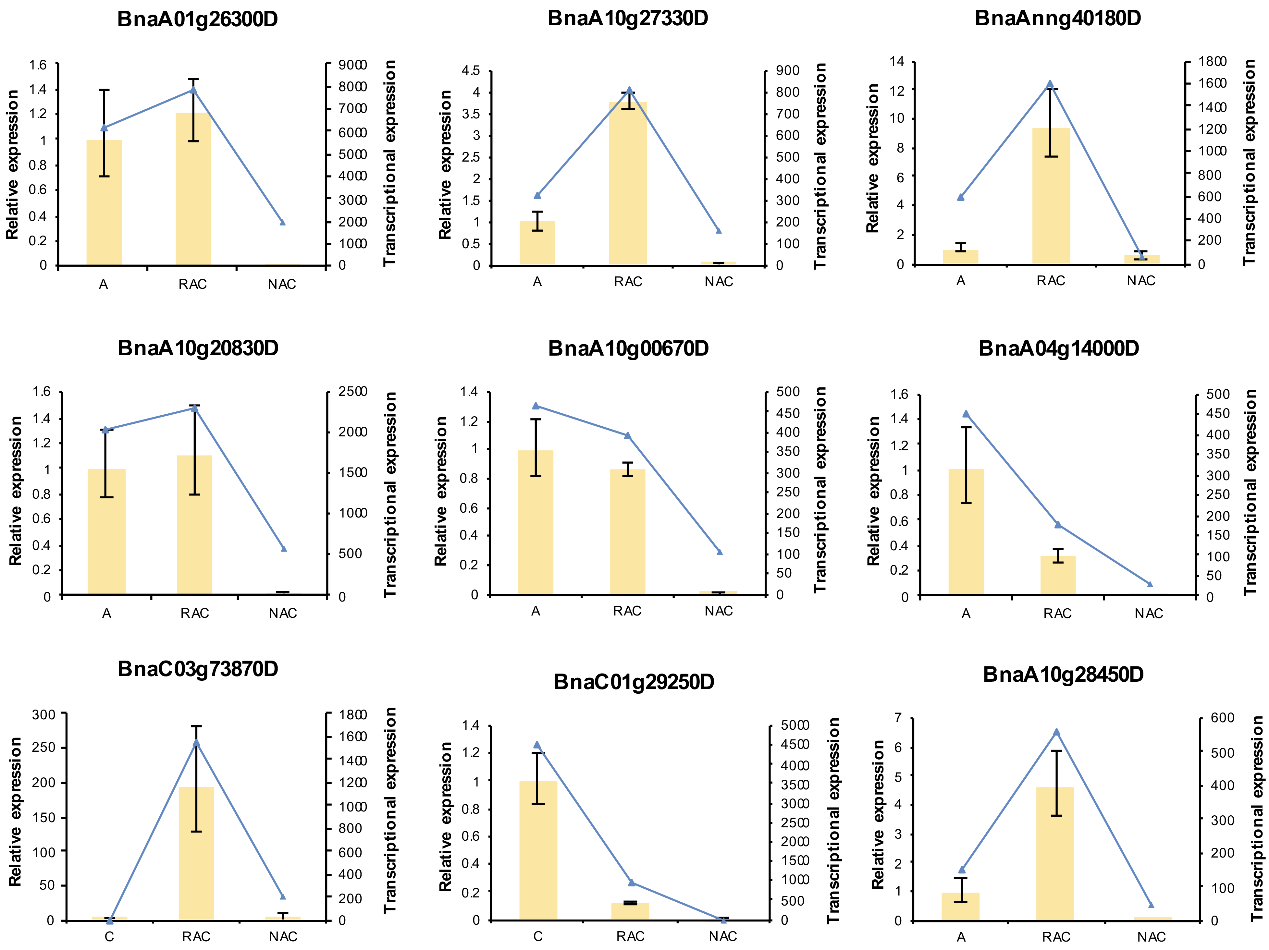


**Figure S1** Relative expression levels of the nine genes verified by qRT-PCR. Yellow columns represent the results from qRT-PCR, and blue dashed lines represent the results from ONT sequencing.

**
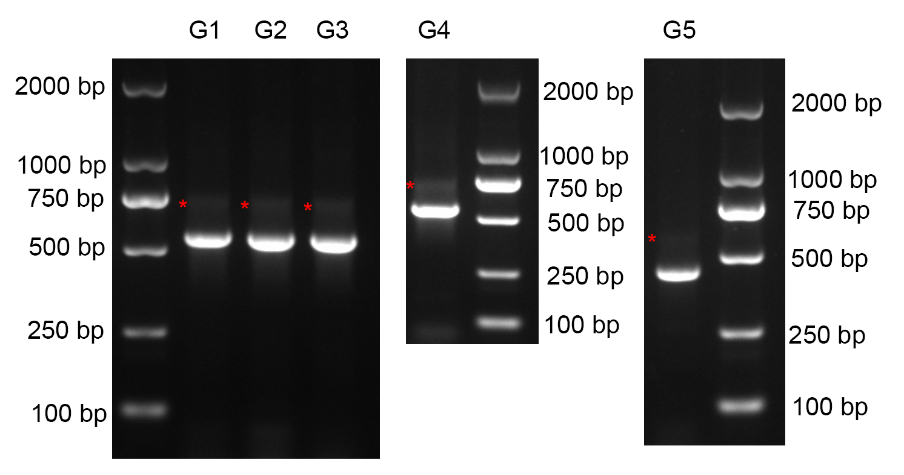
**

**Figure S2** Experimental validation of the alternative splicing events. The maker * indicates bands with low expression levels. G1-G5 represents genes BnaA04g08570D, BnaAnng36210D, BnaC04g30810D, BnaA04g27920D and BnaA08g30780D, respectively.

a

b

c

d

e

f


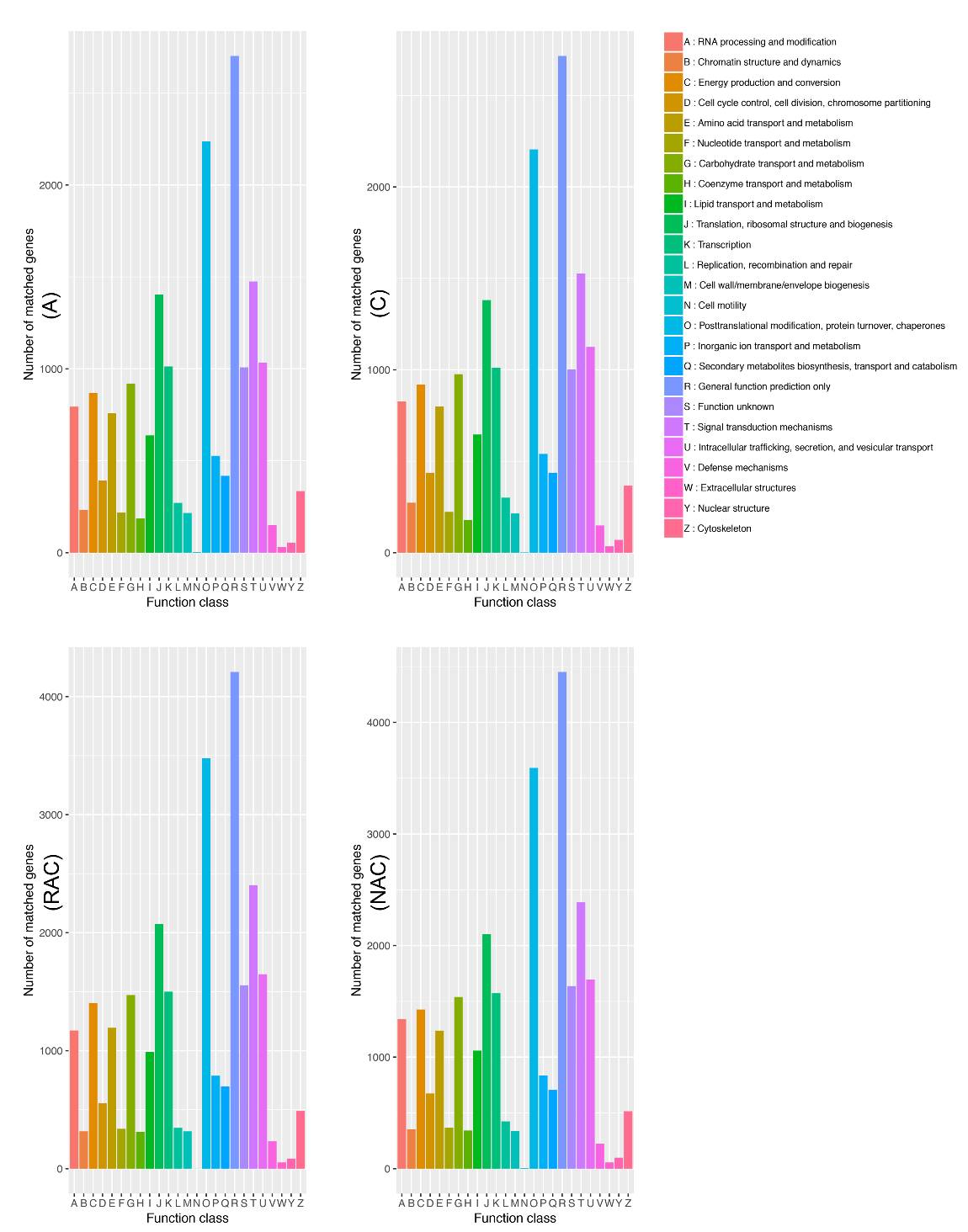


**Figure S3** The functional annotation of all identified isoforms. **a** Functional annotation of isoforms by NR database. **b-e** The GO annotation of isoforms in *B. rapa* (**b**), *B. oleracea* (**c**), resynthesized *B. napus* (**d**) and natural *B. napus* (**e**). **f** The COG annotation of isoforms.

**Table S1** The detection results of RNA sample

| **Sample^a^** | **28S/18S** | **OD_260/280_** | **RNA concentration (ng/µl)** | **RIN value** |
| --- | --- | --- | --- | --- |
| A1 | 1.36 | 2.043 | 984 | 8.5 |
| A2 | 1.32 | 2.017 | 954 | 6.8 |
| A3 | 1.18 | 1.995 | 1260 | 8 |
| C1 | 1.02 | 2.012 | 1220 | 6.9 |
| C2 | 0.83 | 1.981 | 1080 | 6.6 |
| C3 | 1.27 | 1.936 | 318 | 6.6 |
| RAC1 | 1.18 | 1.986 | 680 | 6.8 |
| RAC2 | 1.38 | 2.047 | 600 | 7 |
| RAC3 | 1.35 | 1.874 | 457 | 6.6 |
| NAC1 | 1.19 | 1.985 | 824 | 6.5 |
| NAC2 | 1.31 | 1.989 | 1120 | 7.8 |
| NAC3 | 1.45 | 1.994 | 792 | 7.6 |

^a^1, 2 and 3 represented three biological replicates

**Table S2** Isoform statistics for all samples

| **Sample^a^** | **Total number from isoforms** | **Isoforms from known genes** | **Novel isoform from known genes** | **Isoforms from novel genes** |
| --- | --- | --- | --- | --- |
| A1 | 17,983 | 7,358 | 9,313 | 1,312 |
| A2 | 13,771 | 4,957 | 7,688 | 1,126 |
| A3 | 14,809 | 6,064 | 7,654 | 1,091 |
| C1 | 15,886 | 4,998 | 9,307 | 1,581 |
| C2 | 15,504 | 5,270 | 8,729 | 1,505 |
| C3 | 16,972 | 6,867 | 8,718 | 1,387 |
| RAC1 | 25,280 | 11,431 | 11,796 | 2,053 |
| RAC2 | 26,182 | 11,894 | 12,208 | 2,080 |
| RAC3 | 26,675 | 12,045 | 12,558 | 2,072 |
| NAC1 | 26,072 | 11,598 | 12,310 | 2,164 |
| NAC2 | 20,324 | 8,947 | 9,627 | 1,750 |
| NAC3 | 25,761 | 11,663 | 12,211 | 1,887 |

^a^1, 2 and 3 represented three biological replicates

**Table S3** Statistics of the number of genes of AS occurred only once in each sample

| **Sample^a^** | **ES** | **MX** | **IR** | **A5** | **A3** | **AF** | **AL** | **Sum** | **Total** | **Sum/Total (%)** |
| --- | --- | --- | --- | --- | --- | --- | --- | --- | --- | --- |
| A1 | 136 | 473 | 10 | 47 | 121 | 684 | 646 | 2117 | 2459 | 86.1 |
| A2 | 85 | 258 | 6 | 39 | 94 | 409 | 406 | 1297 | 1482 | 87.5 |
| A3 | 114 | 385 | 5 | 41 | 109 | 500 | 474 | 1628 | 1862 | 87.4 |
| C1 | 113 | 244 | 12 | 50 | 91 | 514 | 519 | 1543 | 1769 | 87.2 |
| C2 | 100 | 188 | 12 | 55 | 87 | 533 | 492 | 1467 | 1649 | 89.0 |
| C3 | 147 | 408 | 13 | 68 | 104 | 680 | 614 | 2034 | 2393 | 85.0 |
| RAC1 | 207 | 531 | 21 | 62 | 178 | 1102 | 916 | 3017 | 3462 | 87.2 |
| RAC2 | 217 | 710 | 17 | 71 | 198 | 1076 | 903 | 3192 | 3658 | 87.3 |
| RAC3 | 235 | 675 | 15 | 73 | 187 | 1097 | 932 | 3214 | 3694 | 87.0 |
| NAC1 | 202 | 549 | 19 | 81 | 182 | 1070 | 918 | 3021 | 3483 | 86.7 |
| NAC2 | 146 | 312 | 12 | 59 | 144 | 761 | 590 | 2024 | 2290 | 88.4 |
| NAC3 | 214 | 514 | 28 | 80 | 178 | 1065 | 920 | 2999 | 3394 | 88.4 |

^a^1, 2 and 3 represented three biological replicates

**Table S4** The forward and reverse primer sequences for qRT-PCR

| Gene ID | Primer | Primer Sequence |
| --- | --- | --- |
| *Actin 2/7* | Forward | TTCAATGTCCCTGCCATGTA |
|  | Reverse | GAGACGGAGGATAGCGTGAG |
| BnaA01g26300D | Forward | ATTAGTGAAGTTTGCCGTCGTTT |
|  | Reverse | GAGTAACTATTCCGGCTACCATCA |
| BnaA10g20830D | Forward | TCCAGCTCCGAGTCCTACGTC |
|  | Reverse | GAAGAGCAGAGCCGAAAACAA |
| BnaAnng40180D | Forward | GTGAAGATGGCAGTCGTTTTTG |
|  | Reverse | CATGAATCCAACGAGAGGAATG |
| BnaA10g28450D | Forward | ATGGATGACAAAACAGGGAAAA |
|  | Reverse | GCGTAACTGGAAAGGCAACAAT |
| BnaA10g00670D | Forward | ACAGGAGGAGGTAAAGCCAAGT |
|  | Reverse | CTGGACGAGACTTGACGATGAA |
| BnaA04g14000D | Forward | GTTGTTGGAACTCTTCGTGGGT |
|  | Reverse | CTTCCACGGTGACAATGCTG |
| BnaA10g27330D | Forward | GATCGTCCTCCGTAATTGTTGC |
|  | Reverse | CTCCTCCCTTCCTCACAAATCC |
| BnaC03g73870D | Forward | AATTAGTGAAGATGGCAGTCGTTT |
|  | Reverse | TCACATGAATCCAACGAGAGGA |
| BnaC01g29250D | Forward | AAGAACATCAACTCAGTCAGCATC |
|  | Reverse | ATGACAGTGTTTCTCTCTTCCCTC |

**Table S5** The forward and reverse primer sequences for PCR amplification in AS validation experiment

| Gene ID | Primer | Primer sequence |
| --- | --- | --- |
| BnaA04g08570D | Forward | ATGGCTTCCTCTATGCTCTCCTC |
|  | Reverse | TTAAGCACCGGTGAAGCTTGG |
| BnaAnng36210D | Forward | ATGGCTTCCTCTATGCTTTCCTC |
|  | Reverse | TTAAGCACCGGTGAAGCTTGG |
| BnaC04g30810D | Forward | ATGGCTTCCTCTATGCTCTCCTC |
|  | Reverse | TTAAGCACCGGTGAAGCTTGG |
| BnaA04g27920D | Forward | ATGGCTTCCTCTATGCTCTCCTC |
|  | Reverse | TTAAGCACCGGTGAAGCTTGG |
| BnaA08g30780D | Forward | ATGGCGATGATGTCAGCATCTTC |
|  | Reverse | TTAAGCTTTAACTTCTTCAATCTCGTC |
